# Analysis of the effect of different withering methods on tea quality based on transcriptomics and metabolomics

**DOI:** 10.3389/fpls.2023.1235687

**Published:** 2023-09-14

**Authors:** Xiaoli Jia, Qi Zhang, Meihui Chen, Yuhua Wang, Shaoxiong Lin, Yibin Pan, Pengyuan Cheng, Mingzhe Li, Ying Zhang, Jianghua Ye, Haibin Wang

**Affiliations:** ^1^College of Tea and Food, Wuyi University, Wuyishan, China; ^2^College of Life Science, Fujian Agriculture and Forestry University, Fuzhou, China; ^3^College of Life Science, Longyan University, Longyan, China

**Keywords:** Wuyi rock tea, withering, gene expression, metabolite, quality

## Abstract

Withering is very important to the quality of Wuyi rock tea. In this study, transcriptomics and metabolomics were used to analyze the effects of different withering methods on tea quality formation. The results showed that sunlight withering (SW) was most beneficial in increasing the gene expression of ubiquinone and other terpenoid-quinone biosynthesis (ko00130), pyruvate metabolism (ko00620), starch and sucrose metabolism (ko00500), and tryptophan metabolism (ko00380) pathways, and increasing the content of nucleotides and derivatives, terpenoids, organic acids and lipids, thus enhancing the mellowness, fresh and brisk taste and aroma of tea. Withering trough withering (WW) was most beneficial in increasing the gene expression of glutathione metabolism (ko00480), phenylpropanoid biosynthesis (ko00940) pathways, increasing the content of phenolic acids and flavonoids, thus enhancing tea bitterness. A comprehensive evaluation of the metabolite content and taste characteristics of tea leaves showed SW to be the best quality and charcoal fire withering (FW) to be the worst quality. This study provided an important basis for guiding the processing of Wuyi rock tea with different flavors.

## 1 Introduction

Wuyi Mountain is one of the most important tea-producing regions in China, mainly producing and making Wuyi Rock Tea. Wuyi rock tea is an oolong tea with a distinctive charm that stems from the rocky crevices in which its tea trees thrive. As we all know, withering is the first and essential step in the premier processing of Wuyi rock tea. The main purpose of withering is to make the tea leaves slowly lose water, soften the leaves and increase the concentration of sap in the cells, which in turn promotes the decomposition of complex compounds into simple ones, forming volatile flavor compounds, amino acids, and monosaccharides, etc., laying the foundation for subsequent tea processing and the formation of special flavors (Ghodake et al., 2006; Deb and Jolvis Pou, 2016).

Wuyi rock tea is harvested and processed from mid-April to mid-May (about 1 month) each year. Traditionally, the processing of Wuyi rock tea relies on sunlight for withering, that is, sunlight causes a slow loss of water from the leaves of the tea tree, which softens the leaves and leads to withering. However, the number of sunny days available for sunlight withering is less than 30% of the annual Wuyi rock tea processing time period. Therefore, there is a saying among the majority of tea farmers that “tea feeds on the sky”, which means that without natural sunlight withering, it is impossible to produce high-quality tea. With the development of technology, a large number of tea withering equipment has been developed by many researchers to cater for non-sunny weather withering, summarized in two main types, namely charcoal fire withering and withering trough withering (Sharma and Dutta, 2018). Charcoal fire withering is done by placing the tea leaves in a hollow circular drum, which is heated by an external charcoal fire and the heated hot air is evenly transferred through a blower to the hollow part of the circular drum, causing the leaves to slowly lose water after being heated, thus completing the withering of the tea leaves. Withering trough withering is to lay the tea leaves flat in a rectangular withering trough and blow the hot air evenly on the leaf layer from the bottom to make the leaves lose water, thus completing the withering of the tea leaves. The withering process of tea leaves also promotes the transformation of substances in the leaves along with the loss of water. Although charcoal fire withering and withering in the trough can promote water loss from tea leaves and complete the withering of tea leaves, it is unclear whether there is a difference between their degree of material transformation and that of sunlight withering, and an in-depth understanding of the effect of different withering methods on tea quality formation is of great significance for the processing of Wuyi rock tea.

During withering, cells in tea leaves are still alive and gene expression is active; therefore, changes in gene expression affect the intensity of different metabolic pathways in tea leaves, which in turn affect metabolic synthesis (Wang et al., 2019; Xu et al., 2020). With the development of transcriptome sequencing and metabolomics technologies, transcriptome sequencing has been used to find significantly changed differential genes and to target key changed metabolic pathways for pathway enrichment, and then metabolite assays have been used to find substances with significantly changed contents in the corresponding metabolic pathways, and then to analyze the effect of changes in gene expression on metabolites between samples (Bjerrum, 2015; Xia et al., 2020a; Chen et al., 2022b; Huo et al., 2023). In particular, the accuracy of transcriptome sequencing analysis has been effectively improved following the release of the tea tree genome, laying an important foundation for further exploration of tea tree gene expression patterns in response to the external environment and their effects on metabolites (Chen et al., 2020; Wang et al., 2020a; Xia et al., 2020b).

Wuyi Mountain, Fujian Province, China is an important tea-producing area in China, belonging to the subtropical region, located at latitude 27°32′36″ to 27°55′15″ north and longitude 117°24′12″ to 118°02′50″ east. The tea-producing area of Wuyi Mountain mainly produces Wuyi Rock Tea, which has special rock charm quality characteristics because its tea trees grow in rock crevices. Rougui tea tree is one of the main varieties of tea trees planted in the Wuyi Mountains and was recognized as a good provincial variety by Fujian Provincial Crop Variety Validation Committee in 1985. There are six main stages in the primary processing of tea leaves, of which withering is the first step in the processing of fresh tea leaves after harvesting. Based on this, the study was conducted to investigate the effects of different withering methods (sunlight withering, charcoal fire withering, and withering trough withering) on the gene expression and metabolites of tea leaves using Rougui, the main tea tree species planted in Wuyi Mountain, to explore the key factors affecting the flavor formation of Wuyi rock tea by different withering methods, with a view to providing some reference for the processing of high-quality Wuyi rock tea.

## 2 Materials and methods

### 2.1 Field experiment and sample collection

In May 2022, this study collected fresh tea leaves (FL) according to the traditional Wuyi rock tea harvesting standard (one bud and three leaves) and treated them in three ways, including sunlight withering (SW), charcoal fire withering (FW) and withering trough withering (WW), with three replicates for each sample. The specific withering method was based on the production process of Wuyi rock tea (Wang et al., 2020b), and was briefly described as follows: sunlight withering with a leaf layer thickness of 5-7 cm, a withering light intensity of 57,000 lux, a withering time of 45 min, and manual turning of tea leaves every 15 min (**Figure S1**); charcoal fire withering with a withering chamber temperature of 35°C, a withering time of 200 min, and manual turning of the leaves every 100 min (**Figure S2**); withering trough withering with a layer thickness of 5-7 cm, a hot air temperature of 35°C, a withering time of 225 min, and manual turning of tea leaves every 75 min (**Figure S3**). After withering in different withering methods, the water content of tea leaves remained basically the same, all between 12% and 13%. Fresh tea leaves and tea leaves after withering in different ways were collected and stored immediately in liquid nitrogen for transcriptional and metabolomic analysis of tea leaves, with three replicates of each sample.

### 2.2 Transcriptome determination of tea tree leaves

Fresh leaves and withered leaves after different withering modes were used for RNA extraction, with three replicates of each sample. RNA extraction from tea tree leaves was performed using TRI reagent (Molecular Research Center, Cincinnati, OH, USA). A 1% agarose gel was used for electrophoresis to detect RNA integrity, and a UV spectrophotometer was used to detect RNA purity and concentration.

The extracted RNA was used for library construction, with the starting RNA being total RNA, greater than 1 μg. NEBNext^®^ UltraTM RNA Library Prep Kit for Illumina^®^ (NEB, USA) was used to construct the library. The specific method was: mRNA with polyA tails was enriched by Oligo(dT) magnetic beads and then randomly interrupted in NEBFragmentation Buffer with divalent cations. Using mRNA fragments as templates and random oligonucleotides as primers, the first strand of cDNA was synthesized in the M-MuLV reverse transcriptase system, followed by degradation of the RNA strand with RNaseH and synthesis of the second strand of cDNA with dNTPs under the DNA polymerase I system. The purified double-stranded cDNA was then end-repaired, added A-tail and sequencing connector, and the cDNA of about 200 bp was screened with AMPure XP beads (Beckman Coulter, Beverly, USA), which were amplified by PCR, and the PCR products were purified again using AMPure XP beads to obtain the final library. After library construction, the libraries were initially quantified using a Qubit 2.0 Fluorometer and diluted to 1.5 ng/μL, followed by an Agilent bioanalyzer 2100 to check the insert size of the library, and after the insert size met expectations, the effective concentration of the library was accurately quantified by *q*RT-PCR to ensure the quality of the libraries.

After the libraries have been assayed and qualified, the different libraries are pooled according to the effective concentration and amount of data coming out of the machine required for the Illumina sequencing target, and 150 bp pairs-end reads are generated (Robinson et al., 2011). The basic principle of sequencing was sequencing while synthesizing. Four fluorescently labeled dNTP, DNA polymerase, and splice primers were added to the sequenced flow cell for amplification. When extending the complementary strand, each addition of the fluorescently labeled dNTP emitted a corresponding fluorescent signal, which was captured by the sequencer and converted into sequencing peaks by computer software to obtain the sequence information of the fragment to be sequenced. Sequence data obtained were analyzed for quality control, i.e. raw data were filtered using fastp to remove the adapter sequence of reads to obtain clean reads for subsequent analysis (Robinson et al., 2011). The clean reads obtained were matched with the reference genome (gcf_004153795.1_ahu_css_1_genomics.fna.gz), and the matching efficiency was analyzed (Zhang et al., 2023). After matching was completed, gene matching was calculated using featureCounts, and then the FPKM of each gene was calculated based on gene length and used to quantify gene expression level (Liao et al., 2014).

### 2.3 Metabolome determination of tea tree leaves

The collected tea leaves were vacuum freeze-dried and ground, 50 mg of tea powder was taken, added to 1200 μL of pre-cooled methanol solution (70%), extracted by vortex shaking, centrifuged and the supernatant was passed through a 0.22 μm filter membrane for metabolite determination in three replicates of each sample.

The sample extracts were determined by ultra performance liquid chromatography-mass spectrometry (UPLC-ESI-MS/MS) system (UPLC, ExionLC™AD; MS, Applied Biosystems 6500 Q TRAP). The liquid chromatographic column was SB-C18 (1.8 µm, 2.1 mm × 100 mm), mobile phase A was pure water containing 0.1% formic acid and mobile phase B was acetonitrile containing 0.1% formic acid. The sample was measured using gradient elution with the basic parameters of 5% mobile phase B at 0 min, increasing linearly to 95% mobile phase B at 9.00 min and maintaining at 95% for 1 min; decreasing to 5% mobile phase B at 10-11 min and equilibrating at 5% until 14 min. The flow rate was set at 0.35 mL/min; the column temperature was 40°C; The injection volume was 2 μL. The electrospray ionization source (electrospray ionization) for mass spectrometry was set at 500°C, the ion spray voltage was 5500 V (positive ionization mode)/-4500 V (negative ionization mode), the ion source gas I, gas II and gas curtain gas were set to 50, 60 and 25 psi respectively and the collision-induced ionization parameters were set to high. Triple quadrupole mass spectrometry scans were performed using multiple reaction monitoring (MRM) and the collision gas (Nitrogen) was set to medium. The declustering potential and collision energy were further optimized. A specific set of MRM ion pairs were monitored in each period, depending on the metabolites eluting in each period.

### 2.4 Statistical analysis

Excel 2017 software was used to perform basic statistics on the data, including calculating mean and variance and making bar charts. Rstudio 3.3 software was used to produce box diagrams, principal component analysis (PCA), volcano diagrams, orthogonal partial least squares-discriminant analysis (OPLS-DA, R library was ropls 0.9.2), KEGG metabolism pathway enrichment diagrams, rose diagrams, TOPSIS analysis, redundancy analysis, and correlation interaction network diagrams (Watkins, 2020).

## 3 Results and discussion

### 3.1 Transcriptome data analysis of tea tree leaves

After Illumina sequencing, a total of 598194260 Mbp of raw reads were obtained from 12 libraries. After filtering, 582580976 Mbp of clean reads were obtained, all accounting for more than 99.97% of the raw reads (**Table S1**), indicating that the results could fully and truly reflect transcriptome changes in tea leaves. The results of the paired efficiency analysis (**Table S2**) showed that the percentage of genes paired to the reference genome by clean reads was above 80%. It can be seen that the reference genome was well assembled and the measured species were consistent with the reference genome, which could be used for further analysis.

### 3.2 Differential gene expression analysis and screening of key genes in tea tree leaves

**Figure 1:**
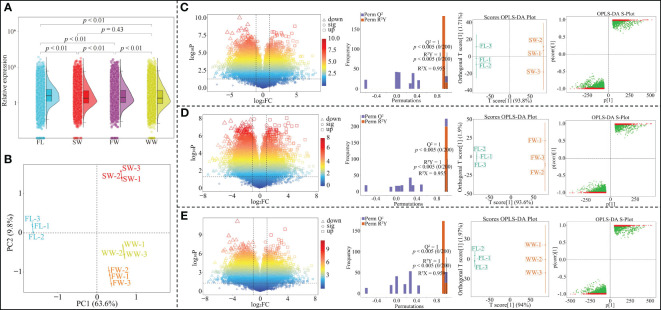
Effect of different withering methods on gene expression in tea leaves and screening of key differential genes. FL, Fresh leaves; SW, Sunlight withering; FW, Charcoal fire withering; WW, Withering trough withering; **(A)** Differential analysis of overall gene expression in tea tree leaves by different withering methods; **(B)** PCA analysis of gene expression in tea leaves by different withering methods; **(C)** Effect of SW on gene expression of tea leaves and screening of key difference genes compared with FL; **(D)** Effect of FW on gene expression of tea leaves and screening of key difference genes compared to FL; **(E)** Effect of WW on gene expression of tea leaves and screening of key difference genes compared to FL.

The results of gene expression analysis of tea tree leaves showed (**Figure 1A**) that the total gene expression of tea tree leaves with different withering methods was significantly different from fresh leaves (*p* < 0.01); secondly, the total gene expression of tea tree leaves after charcoal fire withering (FW) was significantly different from sunlight withering (SW) and withering trough withering (WW) (*p* < 0.01), while there was no significant difference between sunlight withering (SW) and charcoal fire withering (*p* = 0.43). The results of the principal component analysis showed (**Figure 1B**) that the two principal components could distinguish between the four samples, with 63.6% of the contribution of principal component 1 and 9.8% of the contribution of principal component 2, and an overall contribution of 73.4%. OPLS-DA can be used to model the correlation between different indexes and samples and to screen for key genes that characterize differences between samples by variable importance projection values (VIP values) (Li et al., 2023b). Meanwhile, in order to test the reliability of the model, the model is usually validated using a permutation test to evaluate the accuracy of the model (Rivera-Pérez et al., 2022). Accordingly, this study further analyzed the effects of different withering methods on gene expression in tea leaves, and the results showed that the expression of 9171 genes in tea leaves changed significantly after SW compared to fresh leaves (FL); the OPLS-DA model was used to screen key genes (model goodness of fit R^2^Y = 1, *p* < 0.005; predictability Q^2 = ^1, *p* < 0.005), and a total of 6349 key genes with VIP > 1 were obtained, of which 1664 were up-regulated and 4685 were down-regulated (**Figure 1C**). Compared with FL, 7561 genes were significantly changed in tea leaves after FW; key genes were screened using the OPLS-DA model (model goodness of fit R^2^Y = 1, *p* < 0.005; predictability Q^2 = ^1, *p* < 0.005), a total of 5193 key genes with VIP greater than 1 were obtained, of which 1502 genes were up-regulated and 3691 genes were down-regulated (**Figure 1D**). Compared with FL, 9526 genes were significantly changed in tea leaves after WW; key genes were screened using the OPLS-DA model (model goodness of fit R^2^Y = 1, *p* < 0.005; predictability Q^2 = ^1, *p* < 0.005), a total of 6607 key genes with VIP greater than 1 were obtained, of which 1695 genes were up-regulated expression and 6607 genes were down-regulated expression (**Figure 1E**). It can be seen that there were significant differences in the effects of different withering methods on gene expression in tea leaves.

### 3.3 Enrichment of the KEGG pathway for key genes and analysis of their gene expression

**Figure 2:**
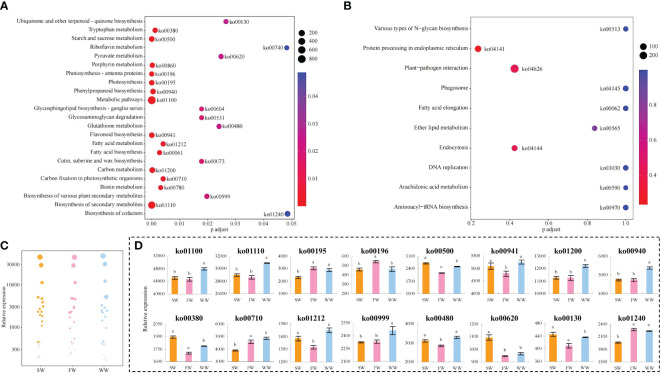
KEGG pathway enrichment and gene expression analysis of key genes in tea leaves by different withering methods. SW, Sunlight withering; FW, Charcoal fire withering; WW, Withering trough withering; **(A)** Enrichment analysis of the KEGG pathway for key genes common to different withering methods; **(B)** Enrichment analysis of the KEGG pathway for differential key genes in different withering methods; **(C)** Screening characteristic KEGG pathway with significant differences in different withering methods; **(D)** Gene expression analysis of characteristic KEGG pathway with significant differences in different withering methods. Lowercase letters indicate significant differences at p<0.05 levels.

On the basis of the above analysis, the study further enriched the key genes in the KEGG pathways, and the results showed (**Figure 2A**) that compared with fresh leaves (FL), 3880 key genes were common and all significantly changed after sunlight withering (SW), charcoal fire withering (FW) and withering trough withering (WW), and a total of 133 KEGG pathways were enriched, of which 23 pathways were enriched to a significant level; secondly, compared with FL, 4476 key genes were different and significantly changed after SW, FW, and WW, and a total of 135 KEGG pathways were enriched, but none of them reached significant levels (*p* > 0.05) (**Figure 2B**, only the first 10 pathways are plotted). In this study, 23 significantly enriched metabolic pathways were used for bubble characteristic analysis, 16 of which significantly changed and were called characteristic pathways (**Figure 2C**). Analysis of gene expression of the characteristic metabolic pathways showed (**Figure 2D**) that for gene expression of 4 characteristic metabolic pathways (ko01100: Metabolic pathways; ko01110: Biosynthesis of secondary metabolites; ko00940: Phenylpropanoid biosynthesis; ko00999: Biosynthesis of various plant secondary metabolites), WW was significantly higher than SW and FW, while the difference between SW and FW was not significant. Phenylpropanoid biosynthesis was a secondary metabolic pathway in plants, and its metabolic capacity was enhanced in the presence of sufficient carbon (Anthony et al., 2023). In this study, the expression of genes in the carbon fixation in photosynthetic organisms (ko00710) pathway was not significantly different between WW and FW, but both were significantly higher than SW; the expression of genes in the carbon metabolism (ko01200) pathway was significantly higher in WW than that of SW and FW, but not different between SW and FW. It is evident that WW is more conducive to improving the metabolic capacity of tea leaves and enhancing the synthesis of its secondary metabolites, especially phenylpropanoid compounds. The products of phenylpropanoid biosynthesis were numerous and included flavonoids, phenols and alkaloids, etc. (Pratyusha and Sarada, 2022; Shu et al., 2022). In the present study, gene expression of the flavonoid biosynthesis pathway (ko00480) was not significantly different between SW and WW, but both were significantly higher than FW. It is assumed that WW was more favorable for the synthesis of flavonoids, phenols and alkaloids in tea leaves, especially flavonoids, followed by SW.

Further analysis revealed that gene expression of the starch and sucrose metabolism (ko00500) pathway was significantly higher in SW than in FW and WW, and significantly higher in WW than in FW. Starch and sucrose metabolism (ko00500) converts large molecules of sugar into small molecules, such as glucose (Li et al., 2022). The catabolism of glucose produces large amounts of intermediate metabolites such as pyruvate and acetyl coenzyme A (Cholico et al., 2023). Pyruvate can be interconverted between sugars, fats and amino acids in the body through acetyl CoA and the tricarboxylic acid cycle (Zhang, 2023). Secondly, acetyl CoA is an essential component of fatty acid and isoprenoid biosynthesis, and is an important precursor for terpenoids and lipids synthesis (Guertin and Wellen, 2023). This study found that gene expression in pyruvate metabolism (ko00620) was significantly higher in SW than in FW and WW, and there were no significant differences between WW and FW. For ubiquinone and other terpenoid-quinone biosynthesis (ko00130) and tryptophan metabolism (ko00380) pathways, gene expression was significantly higher in SW than in FW and WW, and significantly higher in WW than FW. Gene expression of fatty acid metabolism (ko01212) and glutathione metabolism (ko00480) pathways showed no significant difference between SW and WW, but both were significantly higher than FW. It is hypothesized that SW was more conducive to increasing starch and sucrose metabolism, which in turn produced large amounts of intermediate metabolites such as pyruvate and acetyl coenzyme A, providing an important basis for the synthesis of terpenoids, lipids and amino acids, thereby increasing the content of these compounds in tea leaves, especially terpenoids, followed by WW.

### 3.4 Metabolomic analysis and screening of key metabolites in tea tree leaves

**Figure 3:**
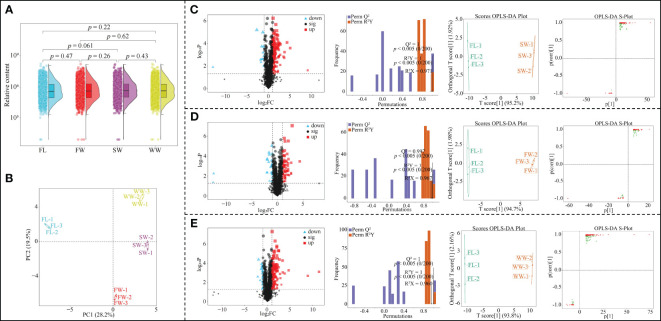
Effect of different withering methods on metabolites of tea leaves and screening of key metabolites. FL, Fresh leaves; SW, Sunlight withering; FW, Charcoal withering; WW, Withering trough withering; **(A)** Analysis of the overall metabolite content of tea leaves by different withering methods; **(B)** PCA analysis of metabolite content of tea leaves by different withering methods; **(C)** Effect of SW on metabolite content of tea leaves and screening of key metabolites compared to FL; **(D)** Effect of FW on metabolite content of tea leaves and screening of key metabolites compared to FL; **(E)** Effect of WW on metabolite content of tea leaves and screening of key metabolites compared to FL.

Based on the above analysis, this study found that different withering methods could significantly affect gene expression in tea leaves, and there were significant differences between different withering methods. To further validate the transcriptomic analysis results, this study analyzed the effects of different withering methods on metabolites in tea leaves using UPLC-ESI-MS/MS technique, and the results showed (**Figure 3A**) that a total of 1897 metabolites were detected in tea leaves in different withering methods (**Figure 3A**); secondly, there was no significant difference in the total amount of metabolites between fresh leaves (FL) and leaves after different withering (SW, FW, WW). The results of principal component analysis based on metabolite content in tea leaves showed (**Figure 3B**) that the two principal components could effectively distinguish between different withering methods, with 28.2% contribution from principal component 1 and 19.5% contribution from principal component 2, with an overall contribution of 47.7%. It can be seen that the total amount of metabolites in tea leaves did not change significantly in different withering methods, but the content of different metabolites was significantly different.

Accordingly, this study further analyzed the effect of different withering methods on the metabolite content of tea leaves. The results showed that compared with fresh leaves (FL), the content of 136 metabolites in tea leaves changed significantly after sunlight withering (SW); the OPLS-DA model was used to screen key metabolites (Model goodness of fit R^2^Y = 1, *p* < 0.005; predictability Q^2 = ^1, *p* < 0.005), and a total of 106 key metabolites with VIP > 1 were obtained, of which 95 showed an increase and 11 a decrease in content (**Figure 3C**). Compared with FL, 85 metabolites changed significantly in tea leaves after FW; the OPLS-DA model was used to screen key metabolites (Model fit R^2^Y = 1, *p* < 0.005; predictability Q^2 = ^0.997, *p* < 0.005), a total of 60 key metabolites with VIP > 1 were obtained, of which 43 increased and 17 decreased in content (**Figure 3D**). Compared with FL, 149 metabolites changed significantly in tea leaves after WW; the OPLS-DA model was used to screen key metabolites (Model goodness of fit R^2^Y = 1, *p* < 0.005; predictability Q^2 = ^1, *p* < 0.005), and 105 key metabolites with VIP > 1 were obtained, of which 92 increased and 13 decreased in content (**Figure 3E**). It can be seen that there were significant differences in the effects of different withering methods on the metabolite content of tea leaves.

### 3.5 Analysis of the content and taste characteristics of characteristic metabolites and their contribution to quality

**Figure 4:**
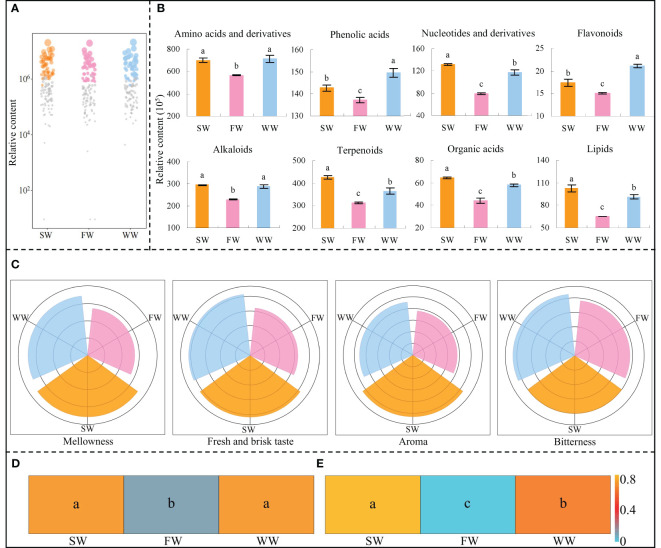
Effects of different withering methods on the content and taste of characteristic metabolites of tea leaves and their contribution to tea quality. SW, Sunlight withering; FW, Charcoal fire withering; WW, Withering trough withering; **(A)** Screening of characteristic metabolites with significant differences of different withering methods; **(B)** Classification and content analysis of characteristic metabolites; **(C)** Analysis of the taste characteristics of characteristic metabolites; **(D)** TOPSIS analysis of the effects of different categories of characteristic metabolites on the overall quality of tea leaves; **(E)** TOPSIS analysis of the effects of different taste characteristics on the overall quality of tea leaves; Lowercase letters indicate significant differences at *p* < 0.05 levels.

Based on the above analysis, the study further analyzed the key metabolites by bubble characteristic map and obtained 47 characteristic metabolites, which could be classified into eight categories of compounds, namely amino acids and derivatives, phenolic acids, nucleotides and derivatives, flavonoids, alkaloids, terpenoids, organic acids and lipids (**Figure 4A**). The results of the content of the different categories of metabolites showed (**Figure 4B**) that the content of amino acids and derivatives and alkaloids in tea leaves was not significantly different between sunlight withering (SW) and withering trough withering (WW), but both were significantly higher than charcoal fire withering (FW); the content of nucleotides and derivatives, terpenoids, organic acids and lipids in tea leaves was significantly higher in SW than in WW and FW, and significantly higher in WW than in FW; the content of phenolic acids and flavonoids in tea leaves was significantly higher in WW than in SW and FW, and significantly higher in SW than in FW. It can be seen that SW was beneficial for the synthesis and accumulation of amino acids and derivatives, alkaloids, nucleotides and derivatives, terpenoids, organic acids and lipids in tea leaves, especially nucleotides and derivatives, terpenoids, organic acids and lipids, followed by WW. WW was beneficial for the synthesis and accumulation of amino acids and derivatives, alkaloids, phenolic acids and flavonoids in tea leaves, especially phenolic acids and flavonoids, followed by SW. The results fully validated the above transcriptomic analysis.

Amino acids and derivatives and nucleotides and derivatives were reported to be important substances in the formation of the fresh and brisk taste of tea leaves, and the higher the content of these substances, the stronger the fresh and brisk taste of tea leaves (Zhang et al., 2019; Huang et al., 2022; Zhang et al., 2022a); Flavonoids and phenolic acids were related to the bitterness of tea leaves, and reducing the content of flavonoids and phenolic acids could reduce the bitterness of tea leaves (Fan et al., 2022; Wang et al., 2023); Organic acids and alkaloids were related to the taste thickness of tea leaves, and increasing their content was beneficial to improve the taste thickness of tea leaves (Wang et al., 2022a; Zhang et al., 2022b); Lipids and terpenoids were important substances for the formation of tea aroma, and the higher their content, the stronger the aroma of tea leaves (Chen et al., 2022a; Hong et al., 2023; Ye et al., 2023). The premier processing of Wuyi rock tea includes several steps. When studying the effect of a processing step on the taste characteristics of tea, numerous scholars often used the metabolites measured in that processing step for the evaluation of taste characteristics (Wang et al., 2019; Wang et al., 2022b; Zhou et al., 2022; Li et al., 2023a). Accordingly, the study further analyzed the effects of different withering methods on the formation of tea taste characteristics based on the content of different categories of compounds, and the results showed (**Figure 4C**, **Table S3**) that SW had the strongest mellowness, fresh and brisk taste and aroma, followed by WW and then FW; while WW had the strongest bitterness, followed by SW and then FW. TOPSIS is a ranking method that approximates the ideal solution, and is a common and effective method in multi-objective decision analysis, which can be ranked by detecting the distance of the evaluation object from the optimal and the worst solution, and it is considered the best if the evaluation object is closest to the optimal solution (Çelikbilek and Tüysüz, 2020). Further, TOPSIS was used to analyze the effect of the combination of different categories of metabolites and taste characteristics on tea quality, and the results showed that there was no significant difference between SW and WW when evaluated comprehensively with different categories of metabolites, but both were significantly higher than FW (**Figure 4D**); when evaluated by different taste characteristics, SW was significantly higher than WW and FW, while WW was significantly higher than FW. It can be seen that SW was the most beneficial method for improving tea quality, followed by WW and then FW (**Figure 4E**).

### 3.6 Correlation analysis of transcriptome and metabolome

**Figure 5:**
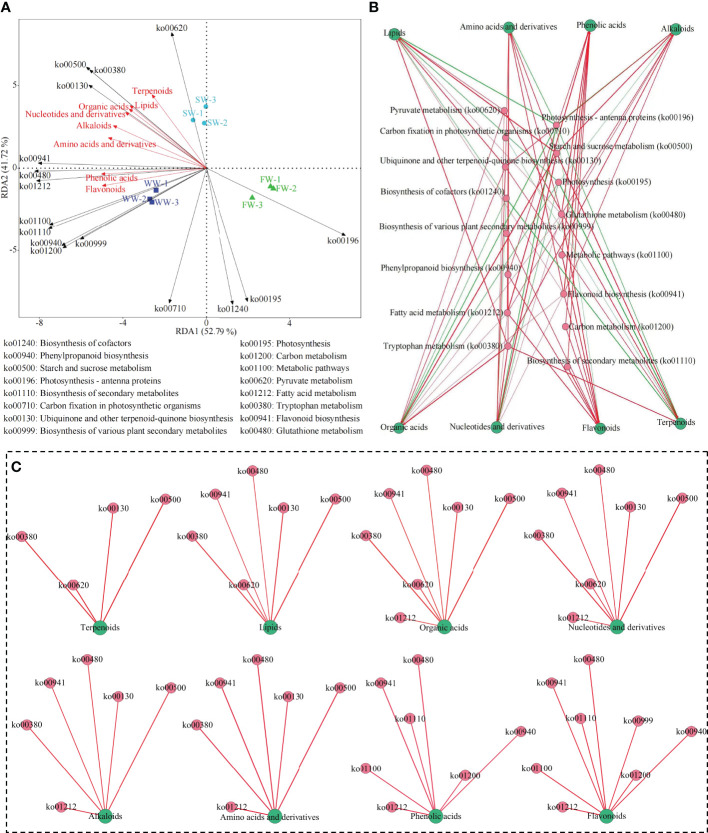
Association analysis between characteristic metabolic pathways and metabolites for different withering methods. SW, Sunlight withering; FW, Charcoal fire withering; WW, Withering trough withering; **(A)** Redundancy analysis of characteristic metabolic pathways and metabolites for different withering methods; **(B)** Total interaction network analysis of characteristic metabolic pathways and metabolites; **(C)** Analysis of interaction networks between characteristic metabolic pathways and different categories of metabolites; ― Represents a significant positive correlation; ― Represents a significant negative correlation.

Based on the above analysis, a joint analysis with gene expression of the characteristic metabolic pathways obtained from the transcriptome and the characteristic metabolites obtained from the metabolome showed (**Figure 5A**) that the main metabolic pathways significantly associated with sunlight withering (SW) were pyruvate metabolism (ko00620), tryptophan metabolism (ko00380), starch and sucrose metabolism (ko00500) and ubiquinone and other terpenoid-quinone biosynthesis (ko00130); the main metabolites significantly associated with SW were terpenoids, lipids, organic acids and nucleotides and derivatives. Secondly, the results of the interaction network analysis (**Figures 5B, C**) showed that the content of terpenoids, lipids, organic acids and nucleotides and derivatives in tea leaves was a significantly positive correlation with the gene expression of ko00620, ko00380, ko00500, and ko00130 pathways after sunlight withering (SW). Wu et al. (2022) analyzed the effects of different withering methods on the formation of aroma of white tea and found that sunlight withering was beneficial to the formation of aroma of white tea. Wang et al. (2022a) analyzed the effects of sunlight withering on the volatile metabolome of tea and concluded that sunlight withering was beneficial to the tea in terms of accumulating and retaining more aroma substances, which was conducive to the enhancement of the aroma of tea. The results validated the suggestion made in the above transcriptomic and metabolomic analysis that SW had the potential to enhance starch and sucrose metabolism in tea leaves, and produced large amounts of intermediate metabolites such as pyruvate and acetyl coenzyme A, and then provided an important basis for the synthesis of terpenoids, lipids and amino acids, which in turn led to the synthesis and accumulation of more terpenoids, lipids, organic acids and nucleotides and derivatives. Further analysis found that (**Figure 5A**) the main metabolic pathways significantly associated with withering trough withering (WW) were flavonoid biosynthesis (ko00941), glutathione metabolism (ko00480), fatty acid metabolism (ko01212), metabolic pathways (ko01100), biosynthesis of secondary metabolites (ko01110), phenylpropanoid biosynthesis (ko00940), carbon metabolism (ko01200) and biosynthesis of various plant secondary metabolites (ko00999). The main metabolites significantly associated with withering trough withering (WW) were phenolic acids and flavonoids. Secondly, the results of the interaction network analysis (**Figures 5B, C**) showed that the content of phenolic acids and flavonoids in tea leaves was significantly positively correlated with the gene expression of ko00941, ko00480, ko01212, ko01100, ko01110, ko00940, and ko01200 pathways after withering trough withering (WW). The results validated the above transcriptomic and metabolomic analysis that suggested WW was more beneficial for the synthesis and accumulation of flavonoids and phenolic compounds in tea leaves.

## 4 Conclusions

**Figure 6:**
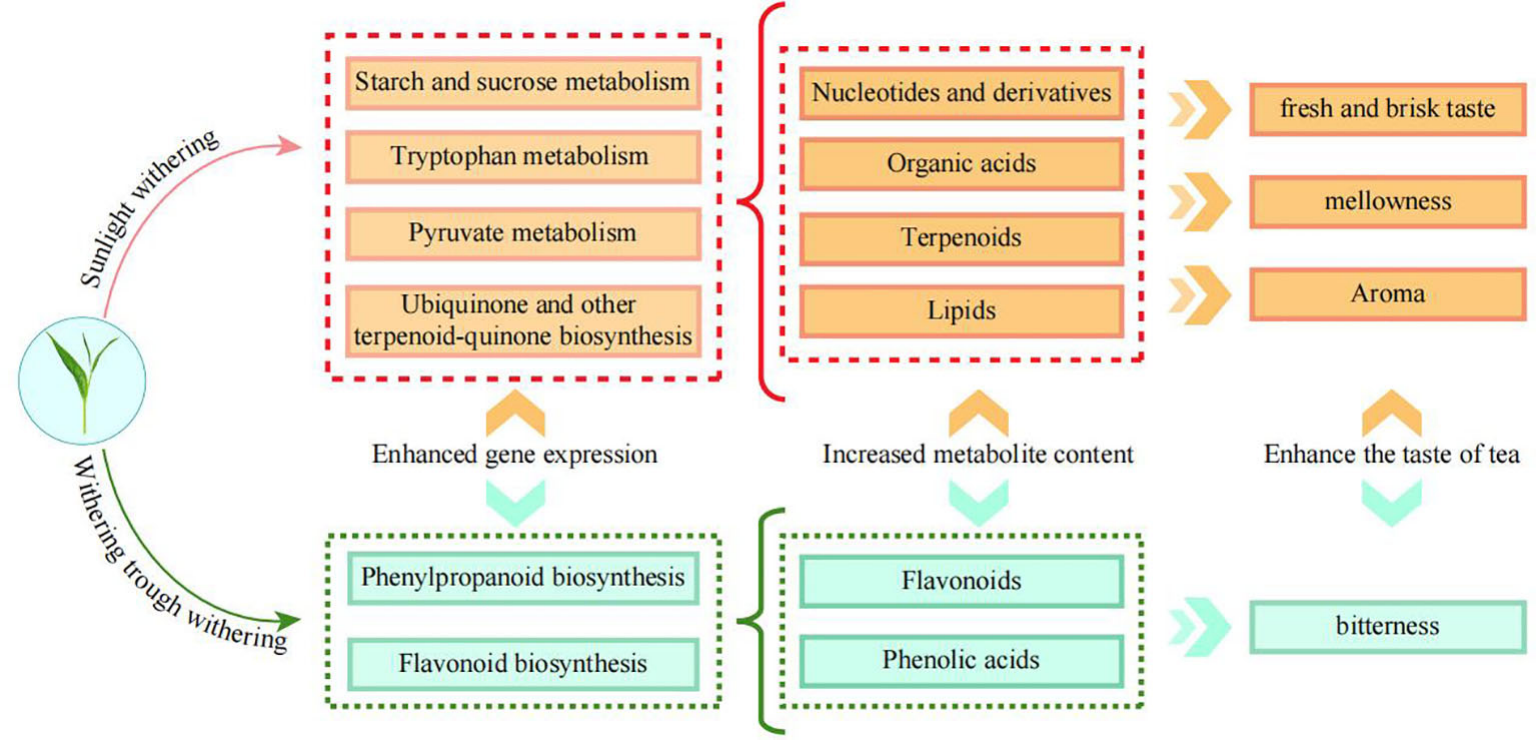
Mechanism analysis of the effect of different withering methods on tea quality.

In this study, the effect of different withering methods on the quality of Wuyi rock tea was analyzed from the perspective of transcriptome and metabolome. It was found (**Figure 6**) that different withering methods significantly affected gene expression and metabolite content of tea leaves, in which SW and WW had the best quality and FW the worst when evaluated comprehensively in terms of key metabolite content; SW had the best quality, WW the second best and FW the worst when evaluated comprehensively in terms of taste characteristics. Among the different withering methods, SW was more conducive to the improvement of gene expression in ubiquinone and other terpenoid-quinone biosynthesis (ko00130), pyruvate metabolism (ko00620), starch and sucrose metabolism (ko00500), and tryptophan metabolism (ko00380) pathways, which were more conducive to the synthesis and accumulation of nucleotides and derivatives, terpenoids, organic acids and lipids in tea leaves, thereby enhancing mellowness, fresh and brisk taste, and aroma of tea leaves. WW was more conducive to the expression of flavonoid biosynthesis (ko00480) and phenylpropanoid biosynthesis (ko00940) pathway genes, and more conducive to the synthesis and accumulation of phenolic acids and flavonoids in tea leaves, thus enhancing bitterness. However, FW was not suitable for the high quality processing of tea leaves. Withering was the first step in the processing of Wuyi rock tea, which was an important foundation for the subsequent processing and quality formation of tea leaves, and this study found that both SW and WW were beneficial to tea processing, but there were some differences in substance transformation, which led to differences in flavor. This study provided an important basis for the production of different flavors of Wuyi rock tea and was of great significance in guiding the processing of Wuyi rock tea.

## Data availability statement

The data presented in the study are deposited in the NCBI repository, accession number PRJNA951194.

## Author contributions

JY and HW conceived the experiments. XJ, QZ, SL and YP performed the experiments. QZ, PC, ML, and YZ analyzed the data. XJ, JY, and HW wrote this manuscript. All authors contributed to the article and approved the submitted version.

## References

[B1] Anthony B. M. Chaparro J. M. Prenni J. E. Minas I. S. (2023). Carbon sufficiency boosts phenylpropanoid biosynthesis early in peach fruit development priming superior fruit quality. Plant Physiol. Bioch. 196, 1019–1031. doi: 10.1016/j.plaphy.2023.02.038 36898214

[B2] Bjerrum J. T. (2015). Metabonomics (New York: Springer).

[B3] Çelikbilek Y. Tüysüz F. (2020). An in-depth review of theory of the TOPSIS method: An experimental analysis. J. Manage. Anal. 7 (2), 281–300. doi: 10.1080/23270012.2020.1748528

[B4] Chen X. Wang P. Wei M. Lin X. Gu M. Fang W.. (2022a). Lipidomics analysis unravels changes from flavor precursors in different processing treatments of purple-leaf tea. J. Sci.Food Agr. 102 (9), 3730–3741. doi: 10.1002/jsfa.11721 34919290

[B5] Chen Y. Zhang S. Du S. Jiang J. Wang G. (2022b). Transcriptome and metabonomic analysis of tamarix ramosissima potassium (K+) channels and transporters in response to NaCl stress. Genes 13 (8), 1313. doi: 10.3390/genes13081313 35893048 PMC9394374

[B6] Chen J. D. Zheng C. Ma J. Q. Jiang C. K. Ercisli S. Yao M. Z.. (2020). The chromosome-scale genome reveals the evolution and diversification after the recent tetraploidization event in tea plant. Hortic. Res. 7, 63. doi: 10.1038/s41438-020-0288-2 32377354 PMC7192901

[B7] Cholico G. N. Orlowska K. Fling R. R. Sink W. J. Zacharewski N. A. Fader K. A.. (2023). Consequences of reprogramming acetyl-CoA metabolism by 2, 3, 7, 8-tetrachlorodibenzo-p-dioxin in the mouse liver. Sci. Rep. 13 (1), 4138. doi: 10.1038/s41598-023-31087-9 36914879 PMC10011583

[B8] Deb S. Jolvis Pou K. R. (2016). A review of withering in the processing of black tea. J. Biosyst. Eng. 41 (4), 365–372. doi: 10.5307/JBE.2016.41.4.365

[B9] Fan F. Y. Zhou S. J. Qian H. Zong B. Z. Huang C. S. Zhu R. L.. (2022). Effect of Yellowing duration on the chemical profile of yellow tea and the associations with sensory traits. Molecules 27 (3), 940. doi: 10.3390/molecules27030940 35164205 PMC8839223

[B10] Ghodake H. M. Goswami T. K. Chakraverty A. (2006). Mathematical modeling of withering characteristics of tea leaves. Dry. Technol. 24 (2), 159–164. doi: 10.1080/07373930600558979

[B11] Guertin D. A. Wellen K. E. (2023). Acetyl-CoA metabolism in cancer. Nat. Rev. Cancer 23, 156–172. doi: 10.1038/s41568-022-00543-5 36658431 PMC11137663

[B12] Hong L. Wang Y. Zhang Q. Wang Y. Chen M. Li M.. (2023). Effects of processing procedures on the formation of aroma intensity and odor characteristic of Benshan tea (Oolong tea, *Camellia sentences*). Heliyon 9, e14855. doi: 10.1016/j.heliyon.2023.e14855 37025800 PMC10070919

[B13] Huang D. Wang Y. Chen X. Wu J. Wang H. Tan R.. (2022). Application of tea-specific fertilizer combined with organic fertilizer improves aroma of green tea. Horticulturae 8 (10), 950. doi: 10.3390/horticulturae8100950

[B14] Huo D. Hao Y. Zou J. Qin L. Wang C. Du D. (2023). Integrated transcriptome and metabonomic analysis of key metabolic pathways in response to cadmium stress in novel buckwheat and cultivated species. Front. Plant Sci. 14. doi: 10.3389/fpls.2023.1142814 PMC10064074 37008482

[B15] Li Z. Sun X. Xu T. Dai W. Yan Q. Li P.. (2023b). Insight into the dynamic variation and retention of major aroma volatile compounds during the milling of Suxiang japonica rice. Food Chem. 405, 134468. doi: 10.1016/j.foodchem.2022.134468

[B16] Li Y. M. You J. L. Nie W. F. Sun M. H. Xie Z. S. (2022). Transcription profiles reveal age-dependent variations of photosynthetic properties and sugar metabolism in grape leaves (*Vitis vinifera* L.). Int. J. Mol. Sci. 23 (4), 2243. doi: 10.3390/ijms23042243 35216359 PMC8876361

[B17] Li Y. Yu S. Yang S. Ni D. Jiang X. Zhang D.. (2023a). Study on taste quality formation and leaf conducting tissue changes in six types of tea during their manufacturing processes. Food Chem. X 18, 100731. doi: 10.1016/j.fochx.2023.100731 37397192 PMC10314197

[B18] Liao Y. Smyth G. Shi W. (2014). featureCounts: an efficient general purpose program for assigning sequence reads to genomic features. Bioinformatics 30 (7), 923–930. doi: 10.1093/bioinformatics/btt656 24227677

[B19] Pratyusha D. S. Sarada D. V. (2022). MYB transcription factors—master regulators of phenylpropanoid biosynthesis and diverse developmental and stress responses. Plant Cell Rep. 41 (12), 2245–2260. doi: 10.1007/s00299-022-02927-1 36171500

[B20] Rivera-Pérez A. Romero-González R. Frenich A. G. (2022). Fingerprinting based on gas chromatography-orbitrap high-resolution mass spectrometry and chemometrics to reveal geographical origin, processing, and volatile markers for thyme authentication. Food Chem. 393, 133377. doi: 10.1016/j.foodchem.2022.133377 35691070

[B21] Robinson J. Thorvaldsdóttir H. Winckler W. Guttman M. Lander E. Getz G.. (2011). Integrative genomics viewer. Nat. Biotechnol. 29 (1), 24–26. doi: 10.1038/nbt.1754 21221095 PMC3346182

[B22] Sharma A. Dutta P. P. (2018). Scientific and technological aspects of tea drying and withering: A review. Agr. Eng. Int. CIGR J. 20 (4), 210–220.

[B23] Shu B. Hu Y. Luo C. (2022). The metabolites involved in phenylpropanoid biosynthesis increase the susceptibility of octoploid strawberry to crown rot caused by *Colletotrichum siamense* . Sci. Hortic. 306, 111447. doi: 10.1016/j.scienta.2022.111447

[B24] Wang X. Feng H. Chang Y. Ma C. Wang L. Hao X.. (2020a). Population sequencing enhances understanding of tea plant evolution. Nat. Commun. 11, 4447. doi: 10.1038/s41467-020-18228-8 32895382 PMC7477583

[B25] Wang P. Gu M. Shao S. Chen X. Hou B. Ye N.. (2022a). Changes in non-volatile and volatile metabolites associated with heterosis in tea plants (*Camellia sinensis*). J. Agr. Food Chem. 70 (9), 3067–3078. doi: 10.1021/acs.jafc.1c08248 35199525

[B26] Wang Y. Li C. Lin J. Sun Y. Wei S. Wu L. (2022b). The impact of different withering approaches on the metabolism of flavor compounds in oolong tea leaves. Foods 11 (22), 3601. doi: 10.3390/foods11223601 36429193 PMC9689020

[B27] Wang H. B. Lin L. W. Wang Y. H. (2020b). Technical specification for tea production, processing and safety inspection (Xiamen, China: Xiamen University Press).

[B28] Wang X. Xiong H. Wang S. Zhang Y. Song Z. Zhang X. (2023). Physicochemical analysis, sensorial evaluation, astringent component identification and aroma-active compounds of herbaceous Peony (*Paeonia lactiflora* Pall) black tea. Ind. Crop Prod. 193, 116159. doi: 10.1016/j.indcrop.2022.116159

[B29] Wang Y. Zheng P. C. Liu P. P. Song X. W. Guo F. Li Y. Y.. (2019). Novel insight into the role of withering process in characteristic flavor formation of teas using transcriptome analysis and metabolite profiling. Food Chem. 272, 313–322. doi: 10.1016/j.foodchem.2018.08.013 30309549

[B30] Watkins M. (2020). A step-by-step guide to exploratory factor analysis with R and RStudio (London, British: Routledge Press).

[B31] Wu H. Chen Y. Feng W. Shen S. Wei Y. Jia H.. (2022). Effects of three different withering treatments on the aroma of white tea. Foods 11 (16), 2502. doi: 10.3390/foods11162502 36010502 PMC9407123

[B32] Xia E. Tong W. Hou Y. An Y. Chen L. Wu Q.. (2020a). The reference genome of tea plant and resequencing of 81 diverse accessions provide insights into its genome evolution and adaptation. Mol. Plant 13, 1013–1026. doi: 10.1016/j.molp.2020.04.010 32353625

[B33] Xia E. Tong W. Wu Q. Wei S. Zhao J. Zhang Z.. (2020b). Tea plant genomics: Achievements, challenges and perspectives. Hortic. Res. 7, 7. doi: 10.1038/s41438-019-0225-4 31908810 PMC6938499

[B34] Xu P. Su H. Zhao S. Q. Jin R. Cheng H. Y. Xu A. A.. (2020). Transcriptome and phytochemical analysis reveals the alteration of plant hormones, characteristic metabolites, and related gene expression in tea (*Camellia sinensis* L.) leaves during withering. Plants 9, 204. doi: 10.3390/plants9020204 32041337 PMC7076645

[B35] Ye J. Wang Y. Lin S. Hong L. Kang J. Chen Y.. (2023). Effect of processing on aroma intensity and odor characteristic of Shuixian (*Camellia sinensis*) tea. Food Chem.: X 17, 100616. doi: 10.1016/j.fochx.2023.100616 36974179 PMC10039254

[B36] Zhang C. (2023). Nutrient metabolism of vertebrate animals. Highlights Sci. Eng. Technol. 30, 85–89. doi: 10.54097/hset.v30i.4957

[B37] Zhang X. Du X. Li Y. Z. Nie C. N. Wang C. M. Bian J. L.. (2022b). Are organic acids really related to the sour taste difference between Chinese black tea and green tea? Food Sci. Nutr. 10 (6), 2071–2081. doi: 10.1002/fsn3.2823 35702304 PMC9179145

[B38] Zhang L. Ho C. T. Zhou J. Santos J. S. Armstrong L. Granato D. (2019). Chemistry and biological activities of processed *Camellia sinensis* teas: A comprehensive review. Compr. Rev. Food Sci. F. 18 (5), 1474–1495. doi: 10.1111/1541-4337.12479 33336903

[B39] Zhang Y. Zhang Q. Wang Y. Lin S. Chen M. Cheng P.. (2023). Effects of magnesium on transcriptome and physicochemical index of tea leaves. Plants 12 (9), 1810. doi: 10.3390/plants12091810 37176867 PMC10181054

[B40] Zhang Q. Zhang Y. Xie J. Ye J. Pang X. Jia X. (2022a). Differences in the analysis of the quality indexes and characteristic amino acids of the different grades of Wuyi Shuixian (*Camellia sinensis*) tea. Food Sci. Technol. 42, e66122. doi: 10.1590/fst.66122

[B41] Zhou C. Chen Z. Li X. Lan C. Xie S. Chen G.. (2022). Transcriptome and phytochemical analyses reveal the roles of characteristic metabolites in the taste formation of white tea during the withering process. J. Integr. Agr. 21 (3), 862–877. doi: 10.1016/S2095-3119(21)63785-1

