# Supplementary material for: Analysis of the effect of different withering methods on tea quality based on transcriptomics and metabolomics
Source: Front Plant Sci. 2023 Sep 14;14:1235687. doi: 10.3389/fpls.2023.1235687 (PMC10538532; doi:10.3389/fpls.2023.1235687)
Supplement: Supplementary file 1 [file DataSheet_1.pdf]

# Supplementary data

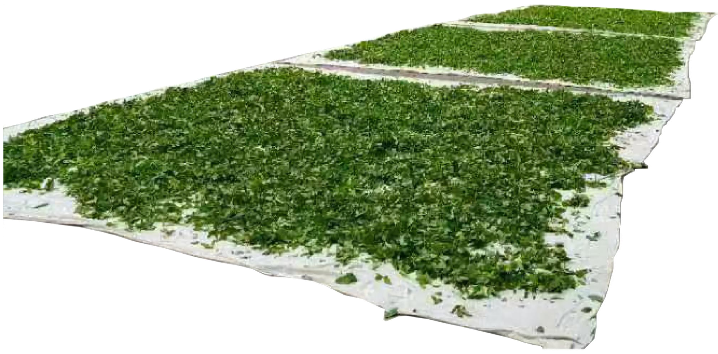

**Figure S1 Sunlight withering**

Note: Sunlight withering mainly relies on natural light to irradiate tea leaves, causing them to lose water and soften, thus achieving the purpose of withering.

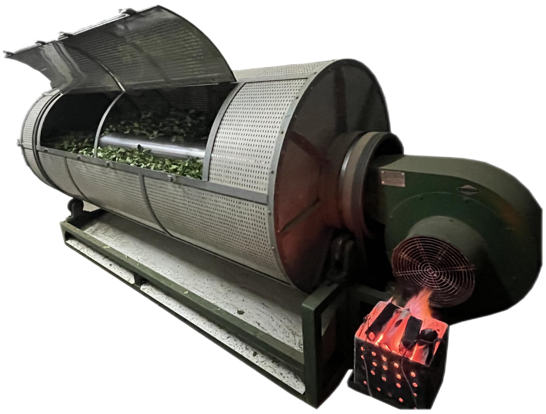

**Figure S2 Charcoal fire withering**

Note: Charcoal fire withering is mainly done by burning charcoal to generated heat and then transferring the hot air through a blower to the tea leaves, causing the leaves to lose water and soften for the purpose of withering.

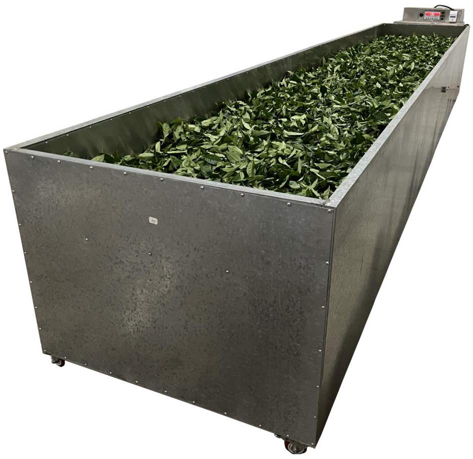

**Figure S3** Withering trough withering

Note: withering trough withering is mainly achieved by blowing hot air at 35°C evenly from the bottom of the tea leaves upwards to soften the leaves by losing water for the purpose of withering.

**Table S1 Overview of the tea leaves metagenomic sequencing**

| Sample | Raw Reads | Clean Reads | Clean Base(G) | Error Rate(%) |
|---|---|---|---|---|
| FL-1 | 46040808 | 44897870 | 6.73 | 0.03 |
| FL-2 | 56987000 | 55603916 | 8.34 | 0.03 |
| FL-3 | 49427916 | 48305008 | 7.25 | 0.03 |
| SW-1 | 48458584 | 47252246 | 7.09 | 0.03 |
| SW-2 | 42778206 | 41612282 | 6.24 | 0.03 |
| SW-3 | 45178478 | 44023788 | 6.60 | 0.03 |
| FW-1 | 44923324 | 43789566 | 6.57 | 0.03 |
| FW-2 | 45770456 | 44618170 | 6.69 | 0.03 |
| FW-3 | 52374362 | 50864110 | 7.63 | 0.03 |
| WW-1 | 60356552 | 58595816 | 8.79 | 0.03 |
| WW-2 | 54889412 | 53355334 | 8.00 | 0.03 |
| WW-3 | 51009162 | 49662870 | 7.45 | 0.03 |
| Total/Average | 598194260 | 582580976 | 87.38 | 0.03 |

Note: SW: Sunlight withering; FW: Charcoal fire withering; WW: withering trough withering.

**Table S2 Efficiency statistics of transcriptome data compared with reference genomes**

| Sample | Total Reads | Reads mapped | Unique mapped | Multi mapped | Read1 mapped | Read2 mapped | '+' mapped | '-' mapped |
|---|---|---|---|---|---|---|---|---|
| FL-1 | 44897870 | 40657563 (90.56%) | 38127642 (84.92%) | 2529921 (5.63%) | 19227985 (42.83%) | 18899657 (42.09%) | 19060822 (42.45%) | 19066820 (42.47%) |
| FL-2 | 55603916 | 50440171 (90.71%) | 47303312 (85.07%) | 3136859 (5.64%) | 23870237 (42.93%) | 23433075 (42.14%) | 23643637 (42.52%) | 23659675 (42.55%) |
| FL-3 | 48305008 | 43841614 (90.76%) | 41056477 (84.99%) | 2785137 (5.77%) | 20702227 (42.86%) | 20354250 (42.14%) | 20519951 (42.48%) | 20536526 (42.51%) |
| FW-1 | 43789566 | 39711899 (90.69%) | 37370719 (85.34%) | 2341180 (5.35%) | 18841996 (43.03%) | 18528723 (42.31%) | 18694557 (42.69%) | 18676162 (42.65%) |
| FW-2 | 44618170 | 40460249 (90.68%) | 38040275 (85.26%) | 2419974 (5.42%) | 19179348 (42.99%) | 18860927 (42.27%) | 19031188 (42.65%) | 19009087 (42.60%) |
| FW-3 | 50864110 | 45868179 (90.18%) | 43158646 (84.85%) | 2709533 (5.33%) | 21822366 (42.90%) | 21336280 (41.95%) | 21588011 (42.44%) | 21570635 (42.41%) |
| SW-1 | 47252246 | 42909824 (90.81%) | 40295091 (85.28%) | 2614733 (5.53%) | 20318830 (43.00%) | 19976261 (42.28%) | 20157640 (42.66%) | 20137451 (42.62%) |
| SW-2 | 41612282 | 37859501 (90.98%) | 35534403 (85.39%) | 2325098 (5.59%) | 17891719 (43.00%) | 17642684 (42.40%) | 17778739 (42.72%) | 17755664 (42.67%) |
| SW-3 | 44023788 | 39967997 (90.79%) | 37529064 (85.25%) | 2438933 (5.54%) | 18901332 (42.93%) | 18627732 (42.31%) | 18777372 (42.65%) | 18751692 (42.59%) |
| WW-1 | 58595816 | 52907053 (90.29%) | 49573077 (84.60%) | 3333976 (5.69%) | 25100495 (42.84%) | 24472582 (41.77%) | 24797684 (42.32%) | 24775393 (42.28%) |
| WW-2 | 53355334 | 48196010 (90.33%) | 45112370 (84.55%) | 3083640 (5.78%) | 22807719 (42.75%) | 22304651 (41.80%) | 22564897 (42.29%) | 22547473 (42.26%) |
| WW-3 | 49662870 | 44777877 (90.16%) | 41720535 (84.01%) | 3057342 (6.16%) | 21115644 (42.52%) | 20604891 (41.49%) | 20862819 (42.01%) | 20857716 (42.00%) |

Note: SW: Sunlight withering; FW: Charcoal fire withering; WW: withering trough withering; The reference genome was GCF_004153795.1_AHAU_CSS_1_genomic.fna.gz. Download address: https://ftp.ncbi.nlm.nih.gov/genomes/all/GCF/004/153/795/GCF_004153795.1_AHAU_CSS_1/; Total Reads: Total number of clean reads; Reads mapped: The number of reads compared to the reference genome; Unique mapped: The number of reads only compared to the reference genome; Multi mapped: The number of reads multiple compared to the reference genome; Read1 mapped: Number of successes comparison in read1; Read2 mapped: Number of successes comparison in read2; '+' mapped: Number of reads successfully comparison on the positive chain of the genome; '-' mapped: Number of reads successfully comparison on the negative chain of the genome.

**Table S3 Analysis of taste characteristics of tea leaves**

| | Relative content ($10^5$) | | | Taste characteristics (References) | Relative content ($10^5$) | | |
|---|---|---|---|---|---|---|---|
| | **SW** | **FW** | **WW** | | **SW** | **FW** | **WW** |
| Alkaloids | 294.72±1.22 | 229.61±1.88 | 288.61±6.99 | Mellowness (Wang et al., 2022; Zhang et al., 2022b) | 359.14±2.37 | 273.52±4.13 | 346.45±7.82 |
| Organic acids | 64.42±1.15 | 43.91±2.26 | 57.84±0.83 | | | | |
| Amino acids and derivatives | 700.91±18.51 | 564.09±4.46 | 711.50±30.31 | Fresh and brisk taste (Huang et al., 2022; Zhang et al., 2019; Zhang et al., 2022a) | 832.37±19.96 | 642.97±5.70 | 828.40±32.42 |
| Nucleotides and derivatives | 131.46±1.45 | 78.88±1.24 | 116.89±2.21 | | | | |
| Terpenoids | 425.94±6.23 | 314.15±4.58 | 364.77±13.92 | Aroma (Chen et al., 2022; Hong et al., 2023; Ye et al., 2023) | 528.70±10.73 | 379.63±4.81 | 456.11±16.57 |
| Lipids | 102.77±4.50 | 65.48±0.23 | 91.34±2.65 | | | | |
| Phenolic acids | 142.75±1.72 | 137.26±1.60 | 149.57±4.89 | Bitterness (Fan et al., 2022; Wang et al., 2023) | 160.15±2.62 | 152.34±1.83 | 170.63±5.30 |
| Flavonoids | 17.40±0.84 | 15.08±0.23 | 21.06±0.41 | | | | |

Note: SW: Sunlight withering; FW: Charcoal fire withering; WW: withering trough withering;

References:

Chen, X., Wang, P., Wei, M., Lin, X., Gu, M., Fang, W., et al. (2022). Lipidomics analysis unravels changes from flavor precursors in different processing treatments of purple-leaf tea. *J. Sci.Food Agr.* 102(9), 3730–3741. doi: 10.1002/jsfa.11721

Fan, F. Y., Zhou, S. J., Qian, H., Zong, B. Z., Huang, C. S., Zhu, R. L., et al. (2022). Effect of Yellowing duration on the chemical profile of yellow tea and the associations with sensory traits. *Molecules* 27(3), 940. doi: 10.3390/molecules27030940

Hong, L., Wang, Y., Zhang, Q., Wang, Y., Chen, M., Li, M., et al. (2023). Effects of processing procedures on the formation of aroma intensity and odor characteristic of Benshan tea (Oolong tea, *Camellia sentences*). *Heliyon* 9, e14855. doi: 10.1016/j.heliyon.2023.e14855

Huang, D., Wang, Y., Chen, X., Wu, J., Wang, H., Tan, R., et al. (2022). Application of tea-specific fertilizer combined with organic fertilizer improves aroma of green tea. *Horticulturae* 8(10), 950. doi: 10.3390/horticulturae8100950

Wang, P., Gu, M., Shao, S., Chen, X., Hou, B., Ye, N., et al. (2022). Changes in non-volatile and volatile metabolites associated with heterosis in tea plants (*Camellia sinensis*). *J. Agr. Food Chem.*70(9), 3067–3078. doi: 10.1021/acs.jafc.1c08248

Wang, X., Xiong, H., Wang, S., Zhang, Y., Song, Z., and Zhang, X. (2023). Physicochemical analysis, sensorial evaluation, astringent component identification and aroma-active compounds of herbaceous Peony (*Paeonia lactiflora* Pall) black tea. *Ind. Crop. Prod.* 193, 116159. doi: 10.1016/j.indcrop.2022.116159

Ye, J., Wang, Y., Lin, S., Hong, L., Kang, J., Chen, Y., et al. (2023). Effect of processing on aroma intensity and odor characteristic of Shuixian (*Camellia sinensis*) tea. *Food Chem.: X*, 17, 100616. doi: 10.1016/j.fochx.2023.100616

Zhang, L., Ho, C. T., Zhou, J., Santos, J. S., Armstrong, L., and Granato, D. (2019). Chemistry and biological activities of processed *Camellia sinensis* teas: A comprehensive review. *Compr. Rev. Food Sci. F.* 18(5), 1474–1495. doi: 10.1111/1541-4337.12479

Zhang, Q., Zhang, Y., Xie, J., Ye, J., Pang, X., and Jia, X. (2022a). Differences in the analysis of the quality indexes and characteristic amino acids of the different grades of Wuyi Shuixian (*Camellia sinensis*) tea. *Food Sci. Technol.* 42, e66122. doi: 10.1590/fst.66122

Zhang, X., Du, X., Li, Y. Z., Nie, C. N., Wang, C. M., Bian, J. L., et al. (2022b). Are organic acids really related to the sour taste difference between Chinese black tea and green tea?. *Food Sci. Nutr.* 10(6), 2071–2081. doi: 10.1002/fsn3.2823
